# Incidental infestations of humans by hard ticks (Acari: Ixodidae) in Colombia: Case reports and record of *Amblyomma cajennense* sensu stricto

**DOI:** 10.1016/j.ttbdis.2025.102565

**Published:** 2025-11-13

**Authors:** Omar Esteban Vargas-Martínez, Luisa Fernanda Naranjo-Vargas, Laura Aramendiz-Macías, Francisco J. Díaz, Juan Carlos Quintero-Vélez, Thiago Fernandes Martins, Juan David Rodas-González

**Affiliations:** a Grupo de Investigación en Ciencias Veterinarias CENTAURO, Facultad de Ciencias Agrarias, Universidad de Antioquia, Medellín 050010, Antioquia, Colombia; b Grupo de Epidemiología, Facultad Nacional de Salud Pública, Universidad de Antioquia, Medellín 050010, Antioquia, Colombia; c Grupo de Mastozoología y Colección Teriológica, Universidad de Antioquia, Medellín 050010, Antioquia, Colombia; d Grupo de Inmunovirología, Facultad de Medicina, Universidad de Antioquia, Medellín 050010, Antioquia, Colombia; e Departamento de Medicina Veterinária Preventiva e Saúde Animal, Faculdade de Medicina Veterinária e Zootecnia, Universidade de São Paulo 05508-270, São Paulo, Brazil

**Keywords:** Ectoparasites, Ixodid, Public health, *Rickettsia*, Vectors

## Abstract

In Colombia, there are few studies that describe tick parasitism in humans, as well as its demographic and ecological characteristics. Additionally, multiple cases of human rickettsiosis caused by highly pathogenic *Rickettsia* species have been reported, whose vector remains unknown. This study reports new cases of human infestation by hard ticks in rural areas of different municipalities of Colombia between 2021 and 2023. The collected ticks were identified using taxonomic keys and molecular and phylogenetic analyses. Likewise, geographical data and other variables associated with parasitism were recorded. A total of 17 hard ticks were collected, associated with 15 infestation cases from nine individuals in eight municipalities across four departments of Colombia. Our findings included infestations by *Amblyomma cajennense* sensu stricto [s.s.] in the department of Meta, a species never reported in Colombia, and *Amblyomma oblongoguttatum* in Antioquia, not reported in humans since 1949. We also report cases of human parasitism by *Amblyomma mixtum, Dermacentor nitens*, and *Rhipicephalus microplus*. No *Rickettsia* bacteria were detected in the ticks. This study highlights the importance of recording the interaction of these ectoparasites with humans in Colombia, expanding knowledge about the diversity of tick species and their parasitic activity in our country.

## 1. Introduction

The ticks are the second most important group of hematophagous arthropods for public health worldwide, since they are biological vectors of a wide variety of infectious agents that affect both humans and domestic and wild animals (Dantas-Torres et al., 2012; Rynkiewicz et al., 2015). Within the Ixodidae family, a total of 785 species of hard ticks have been described globally, associated with different hosts (Guglielmone et al., 2023; Martins et al., 2024). Although humans are not considered natural hosts of these ectoparasites, they are frequently infested during outdoor activities (Guglielmone and Robbins, 2018).

In Colombia, at least 40 species of hard ticks have been identified through molecular and morphological analyses (Acevedo-Gutiérrez et al., 2020; Benavides-Montaño et al., 2022; Cardona-Romero et al., 2020; Cotes-Perdomo et al., 2022; Guglielmone et al., 2021). Among them, 13 ixodid species have been reported infesting humans: *Amblyomma dissimile, Amblyomma mixtum, Amblyomma patinoi, Amblyomma oblongoguttatum, Amblyomma ovale, Amblyomma rotundatum, Amblyomma sabanerae, Amblyomma varium, Dermacentor imitans, Dermacentor nitens, Ixodes tropicalis, Rhipicephalus microplus*, and *Rhipicephalus sanguineus* sensu lato [s.l.] (Álvarez-Londoño et al., 2024; Chitimia-Dobler et al., 2024; Guglielmone et al., 2021; Quintero et al., 2021a, 2017, 2021b).

These findings are highly relevant for the country, given the multiple cases and outbreaks of rickettsiosis in humans, including fatal cases caused by *Rickettsia rickettsii*, for which the vector(s) in Colombia remain (s) unknown (Acosta et al., 2006; Arboleda et al., 2020; Betancourt et al., 2008; Gómez-Quintero et al., 2017; Patiño et al., 2006; Quintero Vélez et al., 2019; Ramírez-García et al., 2021). In addition, there is limited information regarding the frequency, distribution, and impact of human parasitism by hard ticks across the country. Therefore, this study reports new cases and geographical distributions of human infestations by ixodid ticks in different regions of Colombia.

## 2. Materials and methods

We collected a group of ticks found incidentally infesting people who were searching for vectors and hosts of zoonotic rickettsial agents and carrying out faunistic characterizations in rural areas of Colombia during 2021 and 2023. Additionally, ticks found crawling on the skin or on people’s clothing were collected. For each case of infestation, we describe the sex, age group, anatomical site of the infestation, geographical location, altitude, life zone according to Holdridge (1971), and date. Photographic records of some of these infestations were also obtained.

Each collected tick was preserved in a vial with 96 % ethanol and stored in the laboratory of CENTAURO group for research on veterinary sciences of the Universidad de Antioquia (Colombia). The collection and processing of the ticks were approved by the Ethics Committee for Animal Experimentation (CEEA) of the same university, as reported in the minutes of the ordinary meeting n°159.

### 2.1. Taxonomical identification

Taxonomical identification of tick species was performed from morphological characters using a stereomicroscope (Nikon C-PS, Tokyo, Japan). Nymphs and adults were identified according to the literature and keys of Barros-Battesti et al. (2006); Martins et al. (2010; 2014); and Nava et al. (2014). Larvae were identified to genus level by morphological characters such as shape of the base of the capitulum, length of the palps, and presence or absence of festoons. All identifications of specimens of the genus *Amblyomma* were confirmed by molecular techniques.

### 2.2. Molecular analysis

Each tick was air-dried in an incubator at 56°C, then frozen in liquid nitrogen and crushed using a sterile mortar. DNA was extracted using the QIAMP DNA Mini Kit 250 (Qiagen^®^, Germany) according to the commercial protocol (Chitimia-Dobler et al., 2024). The quality and concentration of all DNA extracts were assessed by spectrophotometry (Implen^™^ NanoPhotometer N60). DNA obtained from ticks of the genus *Amblyomma* was used to perform genetic identification by conventional PCR using the constitutive 12S mitochondrial ribosomal (rRNA) gene according to the protocol of Beati and Keirans (2001). The amplification products with non-specific bands on electrophoresis were subjected to DNA purification from agarose gel using the QIAquick Gel Extraction - Gel Purification Kit (Qiagen^®^, Germany). Then, an electrophoresis was performed to assess the presence and purity of each band. Similarly, a conventional PCR was performed on all samples to detect *Rickettsia* DNA, targeting a fragment of the citrate synthase gene (*gltA*) (Labruna et al., 2004).

All amplification products obtained from the constitutive 12S gene were sequenced by the Sanger method (Macrogen^®^, Seoul, Korea). The resulting sequences were labeled according to the corresponding infestation case (EV), assembled using SeqMan^®^ software (DNAStar, USA), and compared with homologous sequences available in GenBank^®^ through BLASTn (NCBI) (Johnson et al., 2008). The verified sequences were subsequently deposited in GenBank^®^.

### 2.3. Phylogenetic analysis

To confirm the tick species of the genus *Amblyomma*, a dataset comprising 130 sequences of the 12S gene from the *Amblyomma cajennense* complex and *A. oblongoguttatum* was constructed. This dataset was aligned with the MUSCLE algorithm and analyzed with the neighbor-joining method in MEGA v11.0.13 (Tamura et al., 2021). The dataset was down sampled eliminating identical or nearly identical sequences from the same country. The final dataset included 61 sequences and was phylogenetically analyzed using the maximum likelihood method in IQ-TREE v. 3.0.1 (Wong et al., 2025). The substitution model used was TIM+F+G4, which was selected using the Model Finder implemented in IQ-TREE. Branch support was estimated using the Ultrafast Bootstrap procedure with 1000 replicates.

## 3. Results

Between 2021 and 2023, a total of 17 Ixodidae ticks were collected, corresponding to 15 infestation events involving nine individuals (designated P1 to P9, Table 1). Two of these events involved two ticks simultaneously attached to the same person and were considered a single case of infestation (Table 1, cases 12 and 15). Four individuals experienced more than one infestation. Fourteen ticks were found attached to the skin, while three were collected from the surface of clothing (Table 1 and Supplementary figure 1). Regarding to people, 67 % were female, and the overall median age was 27 years (IQR: 26 to 38.5). The cases were recorded in eight municipalities across four departments of Colombia: Antioquia (Turbo, Mutatá, Puerto Berrío, San Juan de Urabá, La Ceja), Arauca (Tame), Córdoba (Ayapel) and Meta (Puerto Gaitán).

**Fig. 1.**
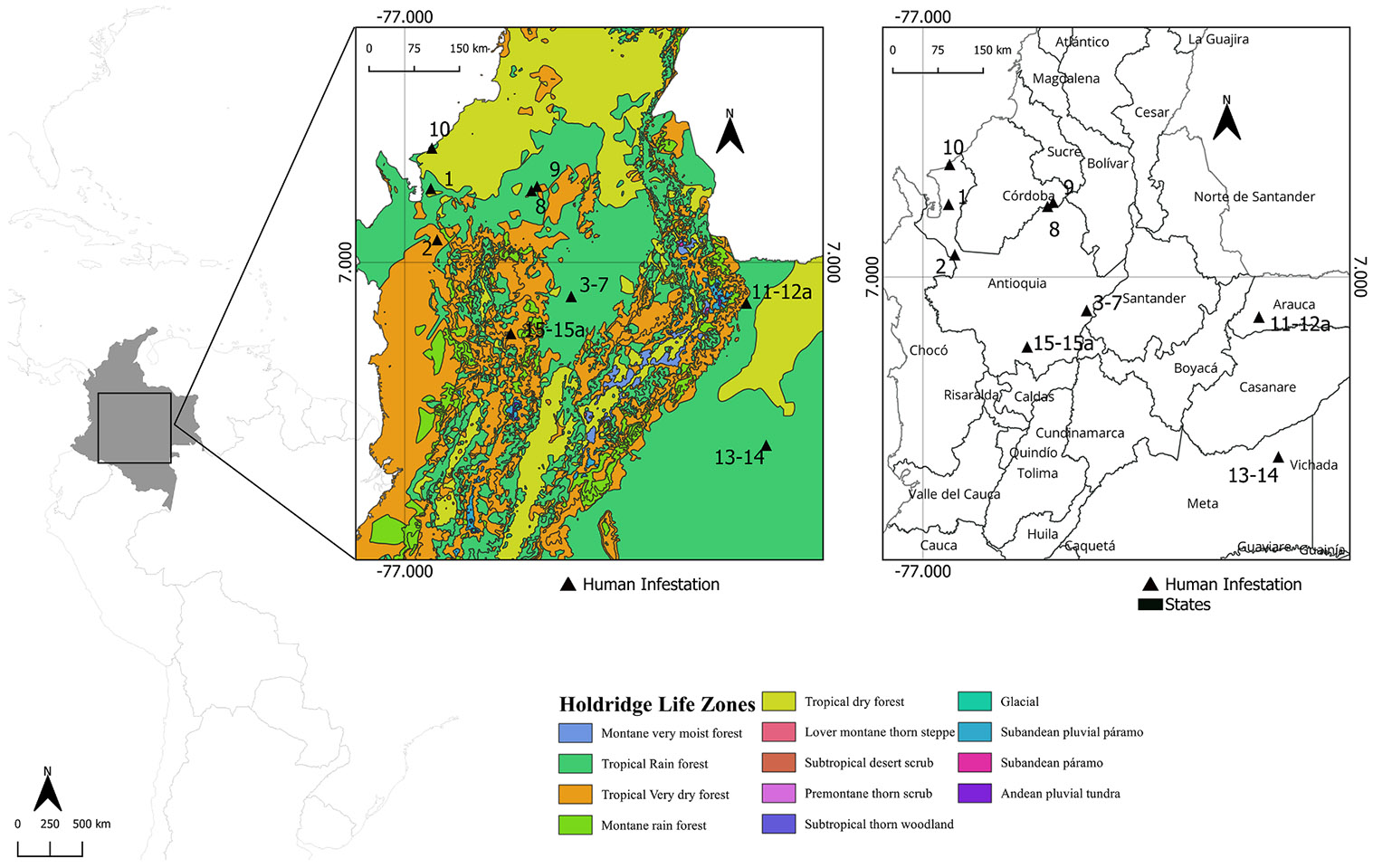
Geographic location of infestation cases in the departments of Córdoba (case N°8–9), Antioquia (case N°1–7, 10, 15–15a), Arauca (case N°11–12a) and Meta (case N° 13–14) in Colombia, with Holdridge life zones.

These cases occurred at different times of the year and at altitudes ranging from 16 to 2333 masl. All recorded cases occurred within the tropical rainforest life zone, specifically within the tropical very humid and tropical very humid low montane categories (Fig. 1). These categories are characterized by average annual temperatures between 24 and 27 °C and annual precipitation levels between 2000 and 4000 mm, depending on the subzone. In addition, the habitat conditions where ticks were collected in association with the cases from Mutatá and Puerto Berrío are potentially related to secondary forest vegetation cover embedded within a mosaic landscape of small relict grasslands and nearby human dwellings. Meanwhile, the ectoparasites collected in the municipality of Tame were clearly associated with a “morichal”, a type of flooded alluvial plain dominated by moriche palms (*Mauritia flexuosa*) and interspersed with scattered shrub vegetation.

The collected ticks were classified as 11 immature individuals (six larvae and five nymphs) and six adults (four males and two females). The identified ixodids belonged to the following species: *Amblyomma cajennense* sensu stricto [s.s.] (one nymph), *A. mixtum* (one nymph; one female; one male), *Amblyomma cajennense* sensu lato [s.l.] (one nymph), *A. oblongoguttatum* (two nymphs; two males), *Amblyomma* sp. (two larvae), *R. microplus* (three larvae; one male), and *D. nitens* (one larva; one female) (Table 1 and Supplementary figure 2). Two of the specimens, *A. oblongoguttatum* (one male) and *R. microplus* (two larvae) were found walking actively on clothing. No specimen tested positive for the *gltA* gene (*Rickettsia* spp.).

**Fig. 2.**
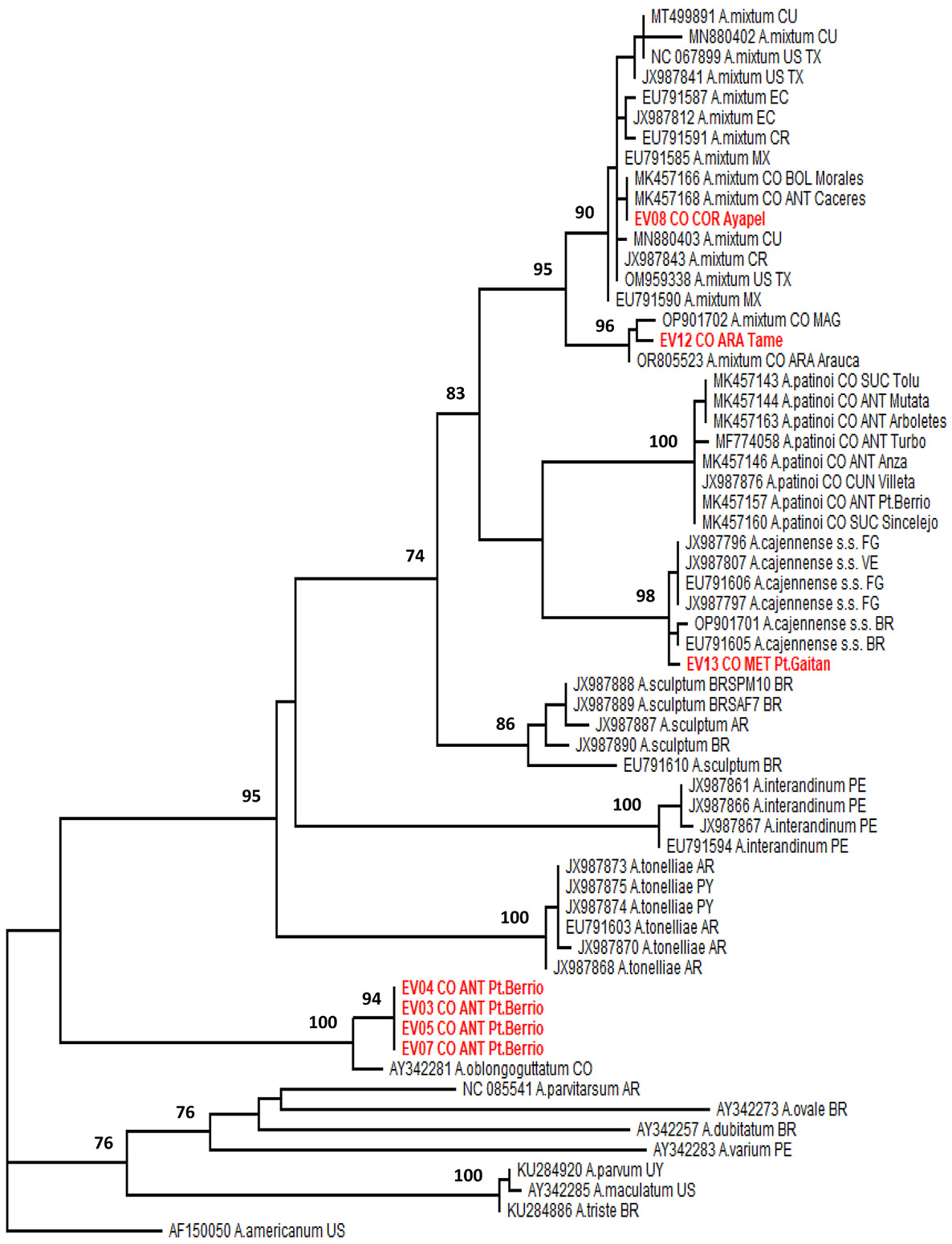
Maximum likelihood phylogenetic tree of constitutive 12S mitochondrial ribosomal (rRNA) gene sequences of species in the *Amblyomma* genus. The sequences obtained in this study (EV) are highlighted in red. The other sequences are named using the GenBank accession number, species name and country of origin in the two-letter international code. The Colombian sequences carry an additional code consisting of the first three letters of the province and the municipality where the ticks were collected. The tree is rooted at the midpoint. Branch supports greater than 70 (ultrafast bootstrap) are shown at the relevant nodes. The analysis was performed in IQ-TREE v.3.0.1 using the TIM+F+G4 model.

In the phylogenetic analysis of the 12S gene (Fig. 2), four of the seven sequences obtained (EV03, EV04, EV05 and EV07) were identical and clustered with the only available *A. oblongoguttatum* sequence, with 100 % support. The other three sequences were polyphyletically distributed within the *A. cajennense* complex: two (EV08 and EV12) grouped in separate subclades of the *A. mixtum* species, and the remaining sequence (EV13) clustered with *A. cajennense* [s.s.] sequences from French Guiana (Roura and Cayenne), Venezuela (Bolívar state), and Brazil (Roraima state), with 98 % support.

## 4. Discussion

This study highlights the record of *A. cajennense* [s.s.] (Puerto Gaitán, Meta, Orinoquia region), which is one of the six tick species that comprise the *A. cajennense* complex. In Colombia, only two species of this complex have been reported, *A. mixtum* and A. *patinoi*, associated with different host species (Acevedo-Gutiérrez et al., 2021; Nava et al., 2014; Rivera-Páez et al., 2016). Therefore, this constitutes a novel record not only for Colombia but also for South America, where the known distribution and reports of human parasitism by this species had been restricted to the Amazon region of Suriname, Venezuela, the Guianas, and the periphery of the Brazilian Amazon biome (Guglielmone and Robbins, 2018; Martins et al., 2016, 2024; Nava et al., 2014).

Another species of the *A. cajennense* complex identified was *A. mixtum*, recorded in the Córdoba and Arauca departments. This tick is recognized as one of the most common species infesting people in South America (Guglielmone and Robbins, 2018) and is considered a potential vector of *R. rickettsii* in Central America (Bermúdez et al., 2016; Labruna et al., 2014). However, although recent studies in Colombia have isolated *Rickettsia amblyommatis* from *A. mixtum* (Chaparro-Gutiérrez et al., 2023), its potential as a vector remains unknown in the country.

On the other hand, adults of *A. oblongoguttatum* have been frequently reported parasitizing humans in the Amazon biome (Labruna et al. 2005; Martins et al., 2017). In Colombia, only one historical case of human parasitism has been reported, which occurred in a forested area of the municipality of Barrancabermeja, Santander department (Luque Forero, 1949). Our study reports new cases of infestation by various stages of this tick species in the municipality of Puerto Berrío, Antioquia department, thereby extending its known geographical distribution. However, additional data on host associations in Colombia is needed (Aguirre et al., 2018; Guzmán-Cornejo et al., 2024; Labruna et al., 2004).

The species *D. nitens* and *R. microplus* are mainly associated with equids and cattle, respectively, and are distributed throughout Colombia (Betancourt et al., 1992; Faccini-Martínez et al., 2016; López et al., 2024; Osorno-Mesa, 2006; Otte, 1990; Rivera-Páez et al., 2018; Segura et al., 2020; Villar et al., 2020). Human parasitism is sporadic, as both species are monoxenous. Larval infestations are associated with human presence in tick-infested environments, whereas nymphs and adults are more commonly linked to the handling of domestic animals, as was observed in this study. *R. microplus* has been reported parasitizing humans across Latin America, from Mexico to Argentina. In other nearby countries, human infestation by *D. nitens* has also been documented, particularly in Bolivia and Venezuela, while reports from Brazil remain unconfirmed (Guglielmone and Robbins, 2018).

Our specimens were collected in a variety of landscapes that overlapped with human settlements and livestock production areas undergoing transitions into wooded environments with varying degrees of anthropogenic disturbance. Thus, Nogueira et al. (2022) mention that human proximity to forested areas, combined with ongoing environmental changes, has contributed to an increase in tick parasitism in humans across multiple Brazilian biomes. These observations emphasize the need for more detailed studies on the distribution and ecology of ixodid species across diverse Colombian ecosystems.

One of the main limitations of this study was the low quality and quantity of DNA extracted from some ticks, which made it impossible to determine the species of certain specimens, restricting their identification to the genus level (*Amblyomma*) or as part of *A. cajennense* [s.l.]. Despite the limited number of reported cases, this study provides valuable information on the diversity of ixodid ticks interacting with humans in the region and includes the first record of *A. cajennense* [s.s.] in the country. This finding underscores the need for further research on the ecology of this species in Colombia. Therefore, we encourage other researchers to report cases of human infestation occurring in the territory, to broaden the current knowledge of this issue.

## Supplementary Material

Figure Supplementary 1 Supplementary figure 1. Hard ticks parasitizing humans: *Amblyomma oblongoguttatum* male on the right wrist (Case 5) (A); *Amblyomma oblongoguttatum* nymph on the left hand (Case 8) (B); engorged *Dermacentor nitens* female on the right leg female (Case 2) (C). A larger view of the tick is provided in the upper right corner of each image.

Figure supplementary 2 Supplementary figure 2. Hard ticks collected parasitizing people: *Amblyomma mixtum* female (case N°12); dorsal view (A), ventral view (B). *Amblyomma oblongoguttatum* male (case N°5); dorsal view (C), ventral view (D). *Amblyomma oblongoguttatum* ninfa (case N°3); dorsal view (E), ventral view (F). *Dermacentor nitens* partially engorged female; (case N°2); dorsal view (G), ventral view (H). Scale measurement 1 mm (mm).

Supplementary material associated with this article can be found, in the online version, at doi:10.1016/j.ttbdis.2025.102565.

## Figures and Tables

**Table 1:** Characterization of hard tick infestation cases in individuals from different departments of Colombia.

N° case	Species of tick	Stage	Date	Municipality	Department	Coordinates	Altitude (masl)	Person / Gender	Body site of infestation
1	*Amblyomma cajennense* sensu lato [s.l.]	N	Feb/021	Turbo	Antioquia	8°6′16″N 76°36′37.6″W	63	P1 / F	Ankle
2	*Dermacentor nitens*	F	Jun/023	Mutatá	Antioquia	7°20′16.6″N 76°30′58″W	70	P2 / F	Leg
3	*Amblyomma oblongoguttatum*	N	Jul/023	Puerto Berrío	Antioquia	6°29′25.1″N 74°30′41.3″W	268	P3 / F	Hip
4	*Amblyomma oblongoguttatum*	M	Jul/023	Puerto Berrío	Antioquia	6°29′25.1″N 74°30′41.3″W	268	P3 / F	On clothes
5	*Amblyomma oblongoguttatum*	M	Jul/023	Puerto Berrío	Antioquia	6°29′25.1″N 74°30′41.3″W	268	P4 / F	Wrist
6	*Rhipicephalus microplus*	M	Jul/023	Puerto Berrío	Antioquia	6°29′25.1″N 74°30′41.3″W	268	P5 / M	Leg
7	*Amblyomma oblongoguttatum*	N	Jul/023	Puerto Berrío	Antioquia	6°29′25.1″N 74°30′41.3″W	268	P5 / M	Hand
8	*Amblyomma mixtum*	N	Sep/023	Ayapel	Córdoba	8°4′23″N 75°6′20.5″W	77	P6 / M	Wrist
9	*Rhipicephalus microplus*	L	Sep/023	Ayapel	Córdoba	8°8′13.6″N 75°1′19.1″W	57	P7 / F	On clothes
10	*Dermacentor nitens*	L	Dec/023	San Juan de Urabá	Antioquia	8°42′32.2″N 76°35′39.3″W	16	P2 / F	Abdomen
11	*Amblyomma* sp.	L	Sep/023	Tame	Arauca	6°23′26.8″N 71°53′34″W	715	P4 / F	Shoulder
12	*Amblyomma mixtum*	F	Sep/023	Tame	Arauca	6°23′26.8″N 71°53′34″W	715	P8 / M	Foot
12a	*Amblyomma mixtum*	M	Sep/023	Tame	Arauca	6°23′26.8″N 71°53′34″W	715	P8 / M	Foot
13	*Amblyomma cajennense* sensu stricto [s.s.]	N	Dec/023	Puerto Gaitán	Meta	4°15′57.6″N 71°35′46.8″W	203	P9 / F	Back
14	*Amblyomma* sp.	L	Dec/023	Puerto Gaitán	Meta	4°15′57.6″N 71°35′46.8″W	203	P4 / F	Abdomen
15	*Rhipicephalus microplus*	L	Feb/023	La Ceja	Antioquia	5°56′9″N 75°24′55″W	2333	P4 / F	Arm
15a	*Rhipicephalus microplus*	L	Feb/023	La Ceja	Antioquia	5°56′9″N 75°24′55″W	2333	P4 / F	On clothes

Abbreviations: M: male; F: female; N: nymph; L: larva; masl: meters above sea level.

## Data Availability

The seven confirmed sequence identifications of *A. cajennense* and *A. oblongoguttatum* specimens from this study were finally submitted to GenBank^®^. The accession codes are PV808211 (EV03), PV808212 (EV04), PV808213 (EV05), PV808214 (EV07), PV808215 (EV08), PV808216 (EV12a), and PV808217 (EV13). No data was used for the research described in the article.
