# Supplementary figures and images for: Incidental infestations of humans by hard ticks (Acari: Ixodidae) in Colombia: Case reports and record of Amblyomma cajennense sensu stricto
Source: Ticks Tick Borne Dis. Author manuscript; Available in PMC 2026 May 22. (PMC13195688; doi:10.1016/j.ttbdis.2025.102565)

## Slide 1
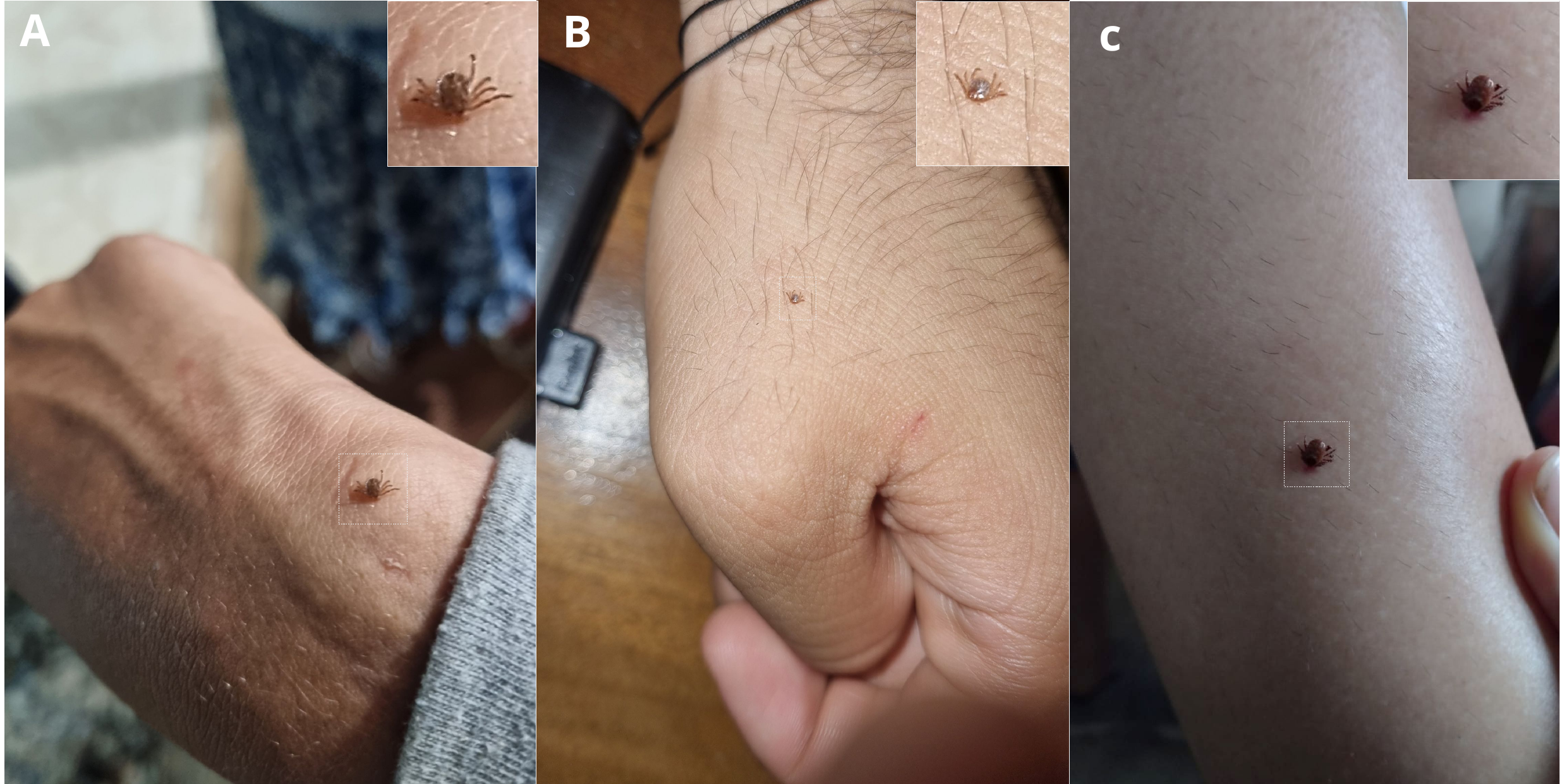

A
B
c

Supplement: Figure Supplementary 1 — Supplementary figure 1. Hard ticks parasitizing humans: Amblyomma oblongoguttatum male on the right wrist (Case 5) (A); Amblyomma oblongoguttatum nymph on the left hand (Case 8) (B); engorged Dermacentor nitens female on the right leg female (Case 2) (C). A larger view of the tick is provided in the upper right corner of each image. [file NIHMS2134730-supplement-Figure_Supplementary_1.pptx]

## Slide 1
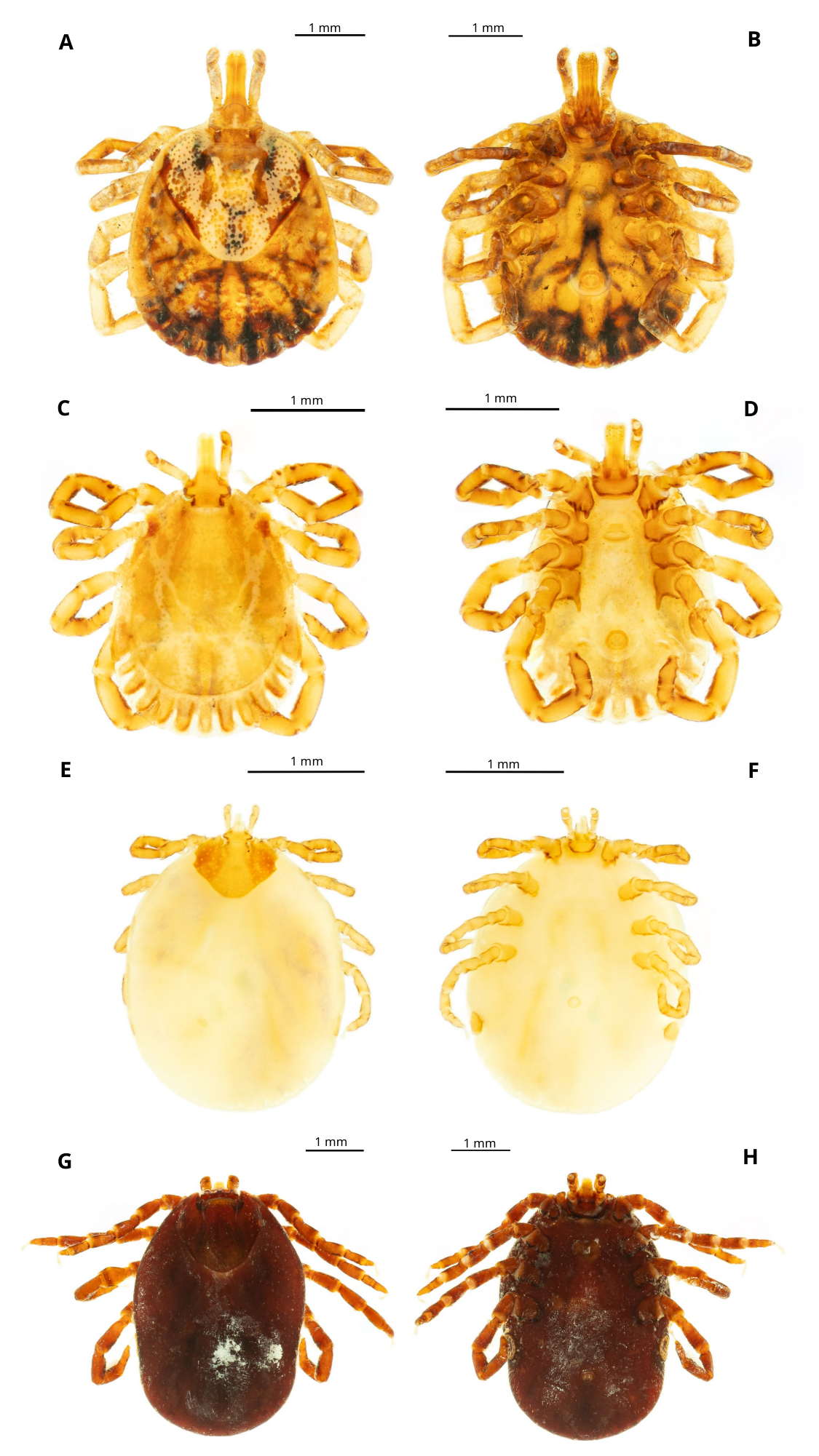

1 mm
1 mm
B
A
1 mm
1 mm
C
D
1 mm
E
1 mm
F
1 mm
1 mm
H
G

Supplement: Figure supplementary 2 — Supplementary figure 2. Hard ticks collected parasitizing people: Amblyomma mixtum female (case N°12); dorsal view (A), ventral view (B). Amblyomma oblongoguttatum male (case N°5); dorsal view (C), ventral view (D). Amblyomma oblongoguttatum ninfa (case N°3); dorsal view (E), ventral view (F). Dermacentor nitens partially engorged female; (case N°2); dorsal view (G), ventral view (H). Scale measurement 1 mm (mm). [file NIHMS2134730-supplement-Figure_supplementary_2.pptx]
